# New Insights on the Role of Allyl Isothiocyanate in Controlling the Root Knot Nematode *Meloidogyne hapla*

**DOI:** 10.3390/plants9050603

**Published:** 2020-05-09

**Authors:** Paul Dahlin, Johannes Hallmann

**Affiliations:** 1 Agroscope, Research Division, Plant Protection, Phytopathology and Zoology in Fruit and Vegetable Production, 8820 Wädenswil, Switzerland; 2 Julius Kühn Institute, Federal Research Centre for Cultivated Plants, Institute for Epidemiology and Pathogen Diagnostics, 38104 Braunschweig, Germany

**Keywords:** biofumigation, isothiocyanate, glucosinolate, plant-parasitic nematodes, root knot nematode, nematicides

## Abstract

Biofumigation, although a well-known method, is still controversially debated as a management strategy for plant-parasitic nematodes (PPN). Its controlling effect is attributed to the production of isothiocyanates (ITCs) following the action of myrosinase on glucosinolates (GSLs). Different ITCs are formed from different GSLs, depending on the plant species. To better understand the potential of ITCs, eight cultivars from three Brassicaceae species were investigated as biofumigation crops to control the root knot nematode *Meloidogyne hapla*. Since results were inconsistent, the nematicidal effect of selected ITCs were further evaluated in vitro. Based on its nematicidal potential, allyl ITC (AITC) was specifically investigated under different soil:sand compositions. A significantly lower nematicidal activity was observed in soil compared to sand. AITC was also evaluated as an additive to the biofumigation in a greenhouse trial. Its supplementation to the biofumigation process with *Brassica juncea* cv. Terrafit controlled *M. hapla*, while no control was observed using *Raphanus sativus* cv. Defender. Thus, the success of biofumigation seems to be strongly dependent on the soil characteristics and the ITC produced during the biofumigation process. Therefore, the supplementation of AITC in combination with the right cover crop can improve the biofumigation process to control *M. hapla*.

## 1. Introduction

Chemical fumigants have been frequently used to protect high value crops from plant-parasitic nematodes (PPN). With stricter legislations and the ban of nematicides harmful to humans and the environment, the search for alternative methods was intensified [1,2,3].

The use of plant-based fumigation appears as an environmentally-friendly management strategy to control PPN [4,5]. This so-called biofumigation makes use of a natural plant defense mechanism of the Brassicaceae family producing the secondary metabolites glucosinolates (GSLs). More than 200 different GSLs are known, which are derived from glucose and amino acids containing sulfur and nitrogen [6,7]. After the damage of the plant tissue, GSLs stored in the vacuoles are hydrolyzed through the enzyme myrosinase (Thioglucosidase: EC 3.2.1.147), stored in myrosin grains (myrosin cells), and then converted into toxic isothiocyanates (ITCs) [7,8]. If, as a result of an attack by pests or pathogens, plant cells are destroyed, ITCs are released and repel or kill the invading organism. Sustainable agriculture systems make use of this chemical reaction by mechanically macerating and incorporating cover crops rich in GSL to induce ITC production for biofumigation [9].

Numerous studies have demonstrated the toxicity of ITCs to PPN [4,5,10] and different effects were observed according to the nematode genus/species [11,12,13]. For example, allyl isothiocyanate (AITC; IUPAC name 3-Isothiocyanatoprop-1-ene) has a higher toxicity against root knot nematodes (*Meloidogyne* spp.) compared to phenyl isothiocyanate. Within *Meloidogyne*, the ITCs butenyl and 4-methylthiobutyl had a higher toxicity against *M. incognita* than *M. javanica* [13,14]. If biofumigation crops are used in praxis to control PPN, results can be contradictory [5,10,15]. Some studies demonstrated significant nematode suppression [16,17], while others showed only minor or no effects on PPN [18,19,20]. An alternative for growing biofumigation crops could be the application of *Brassica*-based seedmeal [21,22] or the use of a biological product containing mustard and chili pepper extracts, with AITC as nematicidal active compound (Dazitol^®^—3.7% AITC) [23]. Both approaches were successfully used to control *M. incognita* and *Pratylenchus penetrans* [21,22].

The nematicidal effect of biofumigation is generally attributed to the effect of the ITCs concentration released by the plants. However, as shown by Vervoort et al. [19] under field conditions, the ITCs concentration is generally too low to account for the nematicidal effect. Besides, no GSL-containing plants express nematicidal effects after maceration and incorporation into the soil, most likely due to unspecific effects associated with the degradation of the organic matter or mechanical disturbance during incorporation. 

Since ITCs are toxic to nematodes in vitro, the question remains how GSL-containing plants can successfully be used to control PPN [4,5]. The objectives of this study were to investigate: (1) the biofumigation performance in the field of different Brassicaceae cultivars on PPN; (2) the effect of soil composition on the efficacy of AITC to control *Meloidogyne hapla*; and (3) if co-application of biofumigation by two *Brassica* species with AITC can enhance control efficacy towards *M. hapla*. In this sense, an accumulated nematicidal effect due to the supplementation of ITC in combination with the right cover crop would potentially foster the biofumigation process to control *M. hapla,* retain plant nutrients and add organic matter to the soil.

## 2. Results

### 2.1. Effect of Biofumigation to Control M. hapla under Field Conditions

The biofumigation process took place at early flowering to flowering depending on the plant species/cultivar and the average fresh weight production ranged between 16.3 and 29.5 kg/m^2^ depending on the Brassicaceae cultivars (Table S1). The total GSL concentration ranged between 10.1 (Terraplus) and 18.9 µmol/g dry matter (Defender) (Table S1). Initial population (Pi) densities of *M. hapla* ranged from 24 (Luna) to 1478 (Terrafit) J2/100 mL of soil and final population (Pf) densities from 36 (Defender) to 1150 (Energy) J2/100 mL of soil (Table 1). This resulted in no significant different multiplication rates (Pf/Pi) varying between 0.72 (Terrafit) and 1.88 (Energy and Luna) despite that Energy and Terrafit have a similar GSL concentration (12.99 and 13.5 µmol/g, respectively) and sinigrin as the most abundant GSL (Table S1).

A reduction in *M. hapla* population (Pf/Pi ˂ 1) was observed for Terrafit, Terraplus, Defender, Adagio, and black fallow. All other cultivars caused an increase in the population density of *M. hapla* (Table 1). 

### 2.2. In Vitro Effect of Different Isothiocyanates on M. hapla Motility

The effect of different commercially available isothiocyanates on *M. hapla* J2 was evaluated in vitro. All ITCs tested significantly reduced J2 activity (Table 2), which was directly related to increasing concentrations and exposure times. The strongest nematicidal activity was observed for benzyl ITC, which caused even at the lowest concentration (0.1 µmol/mL) 100% inactivity within 24 h. The second most active compound was phenyl ITC with 95.8 % inactivity, followed by butyl ITC and AITC with 74.2% and 72.3% inactivity after 24 h of exposure. Except for butyl ITC, all tested ITCs caused complete inactivity following 24 h exposure at the highest concentration (10 µmol/mL). Regarding the effect of increasing concentrations, 2-phenylethyl ITC showed the lowest effect on J2 with 59.3% inactivity after 3 h at 10 µmol/mL (Table 2). 

Following the various treatments, J2 were washed and incubated in water for 24 h. Significant recovery of J2 was observed for butyl ITC, ethyl ITC, methyl ITC, and phenyl ITC at 0.1 and 1 µmol, but not at 10 µmol. The nematodes treated with 0.1 µmol/mL of butyl ITC showed the strongest recovery effect with a reduction from 50.8% to 15.3% inactive J2 after 3 h of exposure, and from 74.2% to 12.8% inactive J2 after 24 h of exposure. In contrast to all other ITCs tested, J2 treated with 2-phenylethyl ITC did not show recovery and J2 inactivity significantly increased after washing and incubation in water (Table 2). 

### 2.3. Long Term In Vitro Effect of Aallyl Isothiocyanate towards M. hapla

All AITC concentrations tested affected *M. hapla* J2 motility in vitro, in comparison to the control (Table 3). After 1, 5, 10, and 20 days, J2 motility was affected by all AITC concentrations and the increase of AITC concentrations caused an increase on the immotility of J2. After one and five days of exposure to 5 and 10 µmol/mL of AITC, J2 motility was affected or J2 were immotile, while, at the highest concentrations of 20 and 40 µmol/mL AITC, all J2 were immotile even at the first day of exposure. The lethal concentrations required to kill 50% (LC_50_) of J2 ranged between 0.19 µmol/mL at Day 5 and 0.07 µmol/mL at Day 7.

In a second approach of this experiment, the AITC treated J2 were inoculated on cucumber seedlings. Three weeks after inoculation, the highest gall index of 6 was obtained in the control for J2 at one day of exposure. With increasing exposure time, the gall index decreased to reach a value of 4 after 20 days of inoculation. J2 exposed to 0.01, 0.1, and 1 µmol/mL of AITC were still able to infect and cause root galling, regardless the number of days of exposure (Table 3). J2 treated with concentrations >5 µmol/mL of AITC did not cause any galling. In comparison to the AITC concentration, the exposure time had little effect on the galling, although there was a slight tendency to gall index decrease with increasing exposure time. 

### 2.4. Effect of Soil Composition on the Control Efficacy of Allyl Isothiocyanate towards M. hapla

To evaluate the effect of soil composition and organic matter content on the nematicidal potential of AITC to control *M. hapla*, we tested different sand:soil mixtures and an organic potting substrate (Table 4). In general, the highest gall index was achieved in the control with values ranging between 4.8 and 5.6 depending on the soil composition (Table 4). With increasing AITC concentrations, gall index was reduced, except for the potting substrate. This effect was most pronounced in sand where concentrations >20 µmol/mL inhibited nematode development. With increasing ratios of soil, the nematicidal effect of AITC was reduced and the gall index increased. For example, the gall index of cucumber roots grown in sand treated with 5 µmol/mL AITC was 2.2 compared to 4.8 in field soil. For soil, only the highest AITC concentration caused a significant reduction in gall index. Besides the control, the overall highest gall index was observed in the organic potting substrate (Table 4). Regardless of the AITC concentration, the gall index ranged between 5.0 (20 and 40 µmol/mL) and 5.4 (10 µmol/mL) and thus AITC does not seem to affect *M. hapla* parasitism under these conditions.

### 2.5. Allyl Isothiocyanate as Additive to the Biofumigation Process to Control M. hapla

Three cover crops were supplemented with different concentrations of AITC at biofumigation and *M. hapla* population density was evaluated two weeks later. The average fresh weight production in the trays were 10.6 kg/m^2^ for Terrafit and 12.2 kg/m^2^ for Defender (Table S2). *M. hapla* densities in the non-treated crops ranged between 81.2 (Defender) and 92.2 (cover crop mix) J2/100 mL soil (Table 5). 

The number of J2 was significantly higher on crops than in the fallow treatment (36.3 J2/100 mL soil) representing the natural decline of *M. hapla*. Supplement of Defender or the cover crop mix with AITC during biofumigation did not result in a reduction of *M. hapla* J2 compared to the non-treated control, regardless of the concentration. In contrast, supplement of Terrafit with AITC at the highest concentration (60 µmol/mL) caused a significant reduction of J2 by 33.4% in comparison to the control. The three crops used for biofumigation did not differ in their effect on *M. hapla* J2 density except at 60 µmol/mL AITC supplement (Terrafit showed the significantly lowest *M. hapla* population density with 57.1 J2/100 mL soil, followed by Defender with 78.5 J2/100 mL soil). 

## 3. Discussion

### 3.1. Effect of Biofumigation to Control M. hapla under Field Conditions

The efficacy of biofumigation to control PPN is discussed controversially among experts. Published reports on biofumigation cover the entire span from successful to no nematode control [4,5]. This is reflected by our own biofumigation data, where *B. juncea* cv. Terrafit caused a reduction in *M. hapla* reproduction, whereas *Sinapis alba* cv. Luna and *B. juncea* cv. Energy did not.

Within the field trial, the content and concentration of GSL produced by the selected plants were in line with previously reported results [19,25,26]. However, Energy supported reproduction of *M. hapla* with a multiplication rate of 1.8, whereas Terrafit had a multiplication rate of 0.72 and thus caused a decrease in *M. hapla* population density. This indicates that the observed reduction of *M. hapla* by Terrafit cannot be explained by its GSL content, since this parameter was similar for both cultivars. 

Nevertheless, it is well documented that the origin and composition of ITCs is relevant for the biofumigation success and can result in crop yield increases up to 30% under optimum conditions [27]. 

### 3.2. In Vitro Effect of Different Isothiocyanates on M. hapla Motility

Generally, the nematicidal activity of the biofumigation process is attributed to the production and the chemical structure (e.g., aliphatic, aromatic, polar, or non-polar) of ITCs, as reported for various PPNs [11,12,13,14,28,29,30,31,32]. According to reports in the literature and confirmed by our results, AITC and benzyl ITC are among the most potent nematicidal ITCs in vitro [12,13,14]. In comparison to AITC and benzyl ITC, 2-phenylethyl ITC showed overall lower nematicidal activity, but it was the only ITC where the nematicidal activity increased even after the J2 were washed in water, indicating an enduring effect. A lower nematicidal activity of 2-phenylethyl ITC in comparison with AITC was also reported *for M. javanica* [13]. 

### 3.3. Long Term In Vitro Effect of Allyl Isothiocyanate towards M. hapla

At the concentration of 0.1 µmol/mL of AITC, significant reduced galling was only observed on Days 1 and 5, suggesting that the weaker J2 were more strongly affected by AITC at onset of the experiment. At 5 µmol/mL of AITC, only affected J2 were observed in vitro, and thus root knot formation did not occur. Therefore, some *M. hapla* might be affected by ITC during the biofumigation process and as a consequence are not infectious in the field, even though those J2 can be evaluated as normal or affected during a short in vitro nematicidal test. Based on the cucumber bioassay, a significant reduction in gall index was achieved at AITC concentrations of 0.1 and 1–5 days of incubation and of 1 µmol/mL or higher, independent of the incubation time. Overall, our AITC lethal concentration at 50% (LC_50_; ranging from 0.07 and 0.19 µmol/mL) were within the range of a previous report for *M. javanica* [12] that measured a LC_50_ value of 0.10 µmol/mL. However, it is important to mention that *M. javanica* J2 were exposed to AITC in sand [12] compared to water in our case, which might have had an influence on the nematicidal effect.

### 3.4. Effect of Soil Composition on the Control Efficacy of Allyl Isothiocyanate towards M. hapla

As is known from the literature, the effect of ITC varies greatly between different studies [4,5]. Major aspects contributing to this variation are assumed to be the soil type and organic matter content. Our results show that AITC activity increased with increasing sand content; this finding is in line with previous findings, where ITCs remained more active in sandy soil than in organic rich soil, or soil with a high clay contend [33,34,35,36,37,38]. Another important aspect to state is that autoclaved soil tends to have a higher AITC recovery than non-autoclaved soil [33,39], suggesting that the microbial community present in the soil plays an important role in the degradation of AITC reducing its half-life. 

Nevertheless, since steamed soil was used in our experiments, we can assume that clay particles and organic matter were the major components inhibiting AITC nematicidal potential, instead of microbial activity.

### 3.5. Allyl Isothiocyanate as Additive to the Biofumigation Process to Control M. hapla

As in the field experiment, the ITCs produced in the tray experiment were not sufficient to control *M. hapla.* Only after AITC was supplemented to the biofumigation, nematode control was achieved for *B. juncea* cv. Terrafit. The difference observed for *B. juncea* cv. Terrafit and *R. sativus* cv. Defender is due to their GSL amount and composition. While *B. juncea* mainly produces sinigrin, the precursor of AITC, *R. sativus* mainly produces 4-methylthiobutyl, the precursor of 4-methylthiobutyl ITC, which might be less effective to control *M. hapla*. Interestingly, according to Lazzeri et al. [14], 4-methylthiobutyl ITC and AITC had a similar immobilizing effect on *M. incognita* with a LD_50_ of 0.021 and 0.034 mM, respectively. However, it seems, that, only if additional AITC is applied to *B. juncea* cv. Terrafit, the AITC concentrations reach levels high enough to control *M. hapla*. Thus, supplementation of AITC during biofumigation with the cover crop *B. juncea* might be an alternative control option for *M. hapla*. 

In the tray experiment, an effect of AITC towards *M. hapla* was only seen at the highest concentration of 60 µmol/mL. This slightly conflicts with results from our previous test in soil where already 40 µmol/mL caused a significant reduction in root gall index caused by *M. hapla*. First, there is always variability in the distribution of AITC within the soil and the two concentrations are not that different. Second, the incorporation of the plant organic matter in the tray experiment might have buffered partly the AITC activity. These assumptions are supported due to the increased application of AITC in the tray experiment compared to the small soil experiment, where no control of *M. hapla* was seen for the cover crop mix and *R. sativus* cv. Defender. Furthermore, steamed soil was used for all the experiments, and, as described by Hanschen et al. [39], autoclaved soil significantly increases AITC half-life. The steamed soil used in the trays had few weeks to increase its bioactivity during the plant growth and watering, compared to the sand:soil experiment and, therefore, the microbial activity established might have had an additional effect on the nematicidal activity of the AITC, as described by Hanschen et al. [39].

### 3.6. Allyl Isothiocyanate as Additive for Biofumigation in the Filed

Our findings raised the question of whether the approach of a biofumigant crop in combination with the direct application of AITC can be used under field conditions. Based on the soil:sand experiment, the nematicidal activity depends on the soil type; the higher is the proportion of sand, the better is the effect. This is confirmed by a biofumigation study with *B. juncea* where a higher amount of AITC was extracted from sandy-loam soil compared to clay–loam soil [33]. Thus, the effect of direct application of AITC in combination with biofumigation on nematode control might be soil type dependent with a clear tendency of better effects in more sandy soils. This is in line with results from field studies in Florida on a Myakka fine sand containing 98% sand, were AITC applications demonstrated good controlling effects on *Meloidogyne* spp. [40]. Those results are of particular interest since sandy soils are generally vulnerable to erosion. However, if biofumigation with the supplementation of AITC is applied during offseason, nematode and erosion control could be easily combined. However, for heavy soils, further research on AITC and other ITCs formulation is needed, to better understand nematode control under those conditions and to find control alternatives.

Since the average fresh weight of the two cultivars Terrafit and Defender was approximately half the amount in the greenhouse compared to the field, the biofumigation process might be more efficient under field conditions because of the higher plant biomass and thus higher ITC rates. Furthermore, as shown by recent kinetic model, ITC formation strongly depends on water availability and pH [41]. To define the optimum conditions, additional investigations to maximize the GSL conversion and to increase the ITCs formation in the field are required. 

If the application of AITC in addition to the biofumigation process proved to be an efficient nematode control alternative, possible formulations and application systems have to be discussed. One option might be biological products containing AITC as the nematicidal active compound, such as Dazitol, showing promising results by controlling *Meloidogyne* spp. in tomato [23,42] and *Globodera* spp. in potato under field conditions [43]. A combination of plant biofumigation and the usage of botanical compounds (e.g., AITC) might have multiple benefits for the soil structure than solely a treatment with AITC. However, due to the broad-spectrum activity of ITCs, it is of utmost importance for all field studies to evaluate its side effects on non-target organisms and the microbiome in general [44,45].

## 4. Material and Methods

### 4.1. Nematode Inoculum and Isothiocyanates

*Meloidogyne hapla* was maintained on tomato (*Solanum lycopersicum*) cv. Moneymaker under greenhouse conditions (25/19 °C, 60% humidity, 15/9 h day night cycle). Freshly hatched second-stage juveniles (J2) were extracted from heavily galled root systems placed under a mist chamber (22 °C) [46]. Hatched J2 were collected every day and stored at 6 °C until being used in experiments, but no longer than 10 days.

The ITCs allyl isothiocyanate, benzyl isothiocyanate, butyl isothiocyanate, ethyl isothiocyanate, methyl isothiocyanate, phenyl isothiocyanate, and 2-phenylethyl isothiocyanate were obtained from Sigma-Aldrich (St. Louis, MO, USA) and diluted with sterilized water to achieve the concentrations requested in the different experiments. For the allyl isothiocyanate (AITC) experiment in soil, the volumetric measure of the pots was used to calculate the concentration (µmol/mL) applied to the soil.

### 4.2. Effect of Biofumigation to Control M. hapla under Field Conditions

The field experiment was conducted at an organic farm with silty loam soil, in Ahlden, Lower Saxony, Germany. The experiment consisted of eight cultivars comprising 3 species from the family Brassicacea: *Raphanus sativus* (RS) cvs. Adagio, Colonel. and Defender; *Brassica juncea* (BJ) cvs. Terrafit, Terraplus. and Energy; and *Sinapis alba* (SA) cvs. Luna and Accent. Each cultivar was replicated 4 times. The biofumigation crops were planted in strips of 50 m × 3 m, and each strip was split into four plots of 12.5 m × 3 m. One strip was kept fallow and used as negative control. Seed density was 12 kg ha^−1^ for *Raphanus sativus* cvs. Adagio, Colonel, and Defender; 15 kg ha^−1^ for *Brassica juncea* cvs. Terrafit, Terraplus, and Energy; and 20 kg ha^−1^ for *Sinapis alba* cvs. Luna and Accent, according to the seed companies’ recommendations. The row distance for all crops was 13 cm.

At harvest, all plants from the central 1 m² of each plot were cut just above the soil surface and fresh and dry weights recorded. From the remaining plants in the plots, 10 plants were randomly selected, uprooted, frozen, and kept at −80 °C. Freeze-dried material was homogenized to powder. A subsample (200 mg) was solubilized in methanol:water (70:30, V:V), at 75 °C and used for GSL extraction in a column stacked with DEAE A25 Sepahdex (CAS Number 12609-80-2, Sigma Aldrich, MO, USA). Converted desulfo-GSLs were used for HPLC-DAD analyzes at a wavelength of 229 nm to evaluate the composition and amount of GSLs produced, as described by Vervoort et al. [19]. GSL composition per hectare was calculated according to GSL concentration and plant biomass.

The biofumigation crops were chopped and incorporated into the top 20 cm of soil, using a tractor-driven flail mower and a rotary tiller. The soil surface was rolled to minimize the evaporation of ITCs. Soil samples were taken prior to planting and four weeks after incorporation of the plants in the soil. Each sample consisted of 30 soil cores per plot, taken from the top 20 cm, using an auger of 2 cm inner diameter. The soil was sieved through a 2 cm mesh to remove stones and plant debris and mix the soil at the same time. Then, 250 mL soil aliquots were processed using the centrifugal flotation method supplemented with kaolin, as described in Hallmann and Subbotin [46]. PPN were counted under a light microscope at 40× magnification. 

### 4.3. In Vitro Effect of Different Isothiocyanates on M. hapla Motility

The inhibition of *M. hapla* by ITCs was tested in vitro in 24-well plates (*n* = 6). Each well was filled with 0.5 mL nematode suspension containing 200 J2 of *M. hapla* plus 0.5 mL of double strength isothiocyanate solutions to result in final concentrations of 0.1, 1, and 10 µmol/mL. All seven ITCs listed above were tested at 20 °C and well plates were kept in dark. Nematode motility was examined after 3 and 24 h under the light microscope at 40× magnification. The first 100 J2 were recorded as active or inactive, then rinsed with tap water, using a 20-µm mesh sieve, and transferred to new 24 well plates containing tap water. Juveniles remaining inactive after 24 h in tap water were considered as dead.

### 4.4. Long Term In Vitro Effect of Allyl Isothiocyanate towards M. hapla 

Because of the high toxicity of AITC and the fact that sinigrin as precursor of AITC occurs at high concentrations in *Brassic juncea*, AITC was selected for further studies on improving the control *M. hapla*. Four thousand *M. hapla* J2 were exposed to 0.01, 0.1, 1, 5, 10, 20, and 40 µmol/mL AITC at 20 °C in dark and evaluated 1, 5, 10, and 20 days after exposure. For each concentration and time point, triplicates of 100 J2 each were evaluated and assigned according to the motility: normal motility, affected motility, and immotility (elongated). From the remaining J2, 4 × 250 J2 from each concentration and time point were rinsed as mentioned above and separately inoculated on pre-germinated cucumber seedlings (*Cucumis sativus* cv. Sprinter F1) in 30 mL pots (*n* = 4). The cucumber plants were grown in a growth chamber at 23 °C ± 2 °C, 16 h photoperiod, and 60% relative humidity. After 21 days of inoculation, cucumber roots were washed and the gall index was determined on a 0–10 scale [24]. 

### 4.5. Effect of Soil Composition on the Control Efficacy of Allyl Isothiocyanate towards M. hapla

The effect of the AITC treatment on *M. hapla* was evaluated using different soil compositions. The original field soil consisted of 4.3% humus, 15.9% clay, 58.7% silt, 25.4% sand, and pH of 6.8 was steamed at 65 °C for 3 h before usage. This soil was mixed with different ratios of silver sand, to obtain sand:soil ratios of 1:0 (100%), 2:1 (67%), 1:1 (50%), 1:2 (33%), and 0:1 (0%). Potting substrate FLORADUR^®^ 10445 (Floragard, Saterland, Germany) containing 90–100% peat was used to represent organic rich soil. Therefore, 60 mL pots (5 replicates/treatment) were filled with 50 mL soil/substrate, watered slightly, and inoculated with 5 mL water suspension containing 250 *M. hapla* J2. After 48 h, 10 mL AITC solutions were added per pot to reach final concentrations of 1, 5, 10, 20, and 40 µmol/mL. The AITC solution was applied on the soil surface and washed into the substrate with 10 mL of water. Finally, the pots were sealed with parafilm for optimum fumigation. After five days, to avoid phytotoxic effects, three-day-old cucumber seedlings were planted into the pots. Twenty-one days after planting, cucumber roots were washed and the root gall index assessed [24].

### 4.6. Allyl Isothiocyanate as Additive to the Biofumigation Process to Control M. hapla

The biofumigant cover crops *Raphanus sativus* (cv. Defender) and *Brassica juncea* (cv. Terrafit) and the non-biofumigant cover crop mix, UFA Maislegum mix (containing *Trifolium hybridum*, *Medicago lupulina, T. incarnatum* and *T. repens*) were grown in 56 cm × 36 cm × 17 cm trays, filled with 25 L of the same (field) soil as described above. The 0.2 m^2^ tray surface was seeded with either 0.6 g seeds of *B. juncea* cv. Terrafit, 1.2 g seeds of *R. sativus* cv. Defender or 0.52 g seeds of the cover crop mix. To mimic different *M. hapla* developmental stages at the time of the biofumigation process (e.g., eggs and J2), seedlings were inoculated with 10,000 and 5000 *M. hapla* J2 at 2 and 3 weeks after sowing, respectively. Two rows, each with 6 holes of 4 cm depth, were distributed over the trays. Each hole was inoculated with 833 J2 in 2 mL water resulting in a total number of 15,000 J2 per pot. After inoculation, trays were watered with a hand nozzle spray. Plants were grown in the greenhouse at 22 ± 2 °C and 16 h photoperiod. The experiment was concluded (32 days post inoculation) after *M. hapla* completed one generation, which was achieved at a greenhouse temperate sum of 450 °C (above 8 °C) [47]. The aboveground plant material was cut and the fresh weight measured. The plant material was then macerated for 15 s by using an electrical string trimmer in a barrel. Macerated plant material was mixed with the soil, using a cement mixer. The soil and plant material mixtures were filled into 60 L plastic bags and placed back into the trays. Four different treatments with final AITC concentrations of 10, 20, 40, and 60 µmol/mL, and a control free of AITC were conducted. To assure that the concentration of AITC was the same for the 25 L of soil, the volumetric amount of the different AITC concentrations was prepared in bottles holding 2.5 L, which was poured to the soil in the plastic bags. Plastic bags were rapidly sealed. Each treatment and control had 6 replicates. Two weeks after AITC treatment, 6 soil cores were taken from each pot, the soil was mixed and the nematodes extracted from 2 aliquots of 100 mL soil using the Oostenbrink dish technique [46]. The total number of extracted *M. hapla* J2 was counted.

### 4.7. Data Analysis

Statistical analyses were performed using the software SPSS 20, and the data tested for homogeneity of variances (Levenes test). The effect of ITCs on *M. hapla* J2 and root gall index data were log_10_(x + 1) transformed. Data were discriminated by one-way ANOVA with post-hoc Tukey HSD (Honestly Significant Difference) test (*p* ≤ 0.05). Differences of independent samples were calculated using a *t*-test. LC_50_ (Lethal concentration, 50%) was determined by polling the data of affected and immotile J2, log-transformed and analyzed by a linear regression model.

## 5. Conclusions

The biofumigation study reflected the inconclusive results regarding nematode control obtained from similar studies. While some Brassicacea successfully controlled *M. hapla*, others supported nematode multiplication. The nematicidal potential of ITCs was clearly demonstrated under in vitro conditions and different ITCs showed different degrees of effectivity. However, in soil systems, the ITC concentration did not seem to be high enough to control *M. hapla*. Only if the *Brassica* cover crop was supplemented with AITC at the time of biofumigation, an effect against *M. hapla* was observed. If application of AITC at time of biofumigation might be an effective method to control *M. hapla* under field conditions still needs to be evaluated.

## Figures and Tables

**Table 1:** Initial (Pi) and final population (Pf) densities and multiplication rates (Pf/Pi) of *Meloidogyne hapla* for eight cultivars comprising three Brassicaceae species, namely *Brassica juncea* (BJ), *Sinapis alba* (SA), and *Raphanus sativus* (RS), in comparison to black fallow included as control (*n* = 4).

Cultivars			Pi	Pf	Pf/Pi
BJ	Energy		612 ± 353	1150 ± 438	1.88
	Terrafit		1478 ± 961	1070 ± 624	0.72
	Terraplus		737 ± 605	567 ± 471	0.77
SA	Luna		24 ± 32	45 ± 15	1.88
	Accent		51 ± 22	54 ± 48	1.06
RS	Defender		39 ± 26	36 ± 42	0.92
	Adagio		140 ± 135	128 ± 83	0.91
	Colonel		171 ± 145	232 ± 99	1.36
		Fallow	87 ± 118	82 ± 105	0.94

**Table 2:** Effect of different isothiocyanates at different concentrations and exposure times on *Meloidogyne hapla* second-stage juvenile’s motility, with and without recovery in water (w.i.).

ITC	Exposure Time	Juvenile Inactivity (%)			
Concentration		H_2_O	0.1 µmol/mL	1 µmol/mL	10 µmol/mL
Allyl ITC	3 h (w.i.)	2.2 ^a1^ (1.9 ^a^)	79.7 ^b1^ (75.0 ^b^)	95.8 ^b1^ (100.0 ^b^)	100.0 ^b1^ (100.0 ^b^)
	24 h (w.i.)	2.3 ^a1^ (2.4 ^a^)	72.3 ^b1^ (77.2 ^b^)	99.8 ^b1^ (100.0 ^b^)	100.0 ^b1^ (100.0 ^b^)
Benzyl ITC	3 h (w.i.)	1.2 ^a1^ (1.2 ^a^)	94.3 ^b1^ (99.8 ^b^)	97.8 ^c1^ (99.7 ^b^)	100.0 ^c1^ (100.0 ^b^)
	24 h (w.i.)	1.2 ^a1^ (1.2 ^a^)	100.0 ^b2^ (100.0 ^b^)	100.0 ^b2^ (100.0 ^b^)	100.0 ^b1^ (97.7 ^b^)
Butyl ITC	3 h (w.i.)	1.2 ^a1^ (2.0 ^a^)	50.8 ^b1^ (15.3 ^b^*)	71.8 ^c1^ (44.5 ^c^*)	87.3 ^d1^ (96.3 ^d^)
	24 h (w.i.)	2.5 ^a1^ (1.8 ^a^)	74.2 ^b2^ (12.8 ^b^*)	86.3 ^c2^ (68.2 ^c^*)	98.7 ^d2^ (99.8 ^d^)
Ethyl ITC	3 h (w.i.)	1.2 ^a1^ (2.0 ^a^)	25.2 ^b1^ (14.3 ^b^*)	91.0 ^c1^ (82.2 ^c^*)	99.3 ^c1^ (99.3 ^d^)
	24 h (w.i.)	2.5 ^a1^ (1.8 ^a^)	27.7 ^b1^ (6.8 ^a^*)	99.2 ^c2^ (57.2 ^b^*)	100.0 ^c1^ (100.0 ^c^)
Methyl ITC	3 h (w.i.)	1.2 ^a1^ (2.0 ^a^)	4.0 ^a1^ (5.7 ^a^)	79.2 ^b1^ (44.2 ^b^*)	99.3 ^b1^ (100.0 ^c^)
	24 h (w.i.)	2.5 ^a1^ (1.8 ^a^)	12.0 ^b2^ (11.8 ^a^)	89.3 ^c2^ (47.3 ^b^*)	100.0 ^d1^ (100.0 ^c^)
Phenyl ITC	3 h (w.i.)	1.3 ^a1^ (1.3 ^a^)	55.5 ^b1^ (52.2 ^b^)	98.0 ^c1^ (90.3 ^c^*)	99.7 ^c1^ (99.3 ^c^)
	24 h (w.i.)	1.0 ^a1^ (2.0 ^a^)	95.8 ^b2^ (73.0 ^b^*)	99.5 ^c1^ (96.0 ^c^*)	100.0 ^c1^ (100.0 ^c^)
2-phenylethyl ITC	3 h (w.i.)	1.3 ^a1^ (1.3 ^a^)	5.7 ^ab1^ (6.8 ^a^**)	21.2 ^b1^ (75.0 ^b^**)	59.3 ^c1^ (99.3 ^c^)
	24 h (w.i.)	1.0 ^a1^ (2.0 ^a^)	16.8 ^b2^ (38.7 ^b^**)	89.8 ^c2^ (98.3 ^c^**)	100.0 ^c2^ (99.8 ^c^)

*M. hapla* second-stage juveniles (J2) response to 0.1, 1, and 10 µmol/mL of different isothiocyanates (ITCs) exposed for 3 and 24 h, followed by nematode washing in water (H_2_O) and a 24 h H_2_O recovery assay. Different superscript letters indicate significantly inactive J2 (movement affected, inhibited, or immobile) in percentage (%) over the concentrations calculated using a one-way ANOVA with post-hoc Tukey HSD test (*n* = 6). Different superscripted numbers indicate significantly inactive J2 in percentage (%) over time (3 and 24 h), while significant H_2_O recovery is indicated by an *, and significant persistent inactivation on J2 after the H_2_O recovery attempt is indicated by **, analyzed by *t*-test. w.i., H_2_O incubation, indicating the recovery assay. *p* < 0.05.

**Table 3:** Effect of allyl isothiocyanate (AITC) on *Meloidogyne hapla* second-stage juveniles (J2) after 1, 5, 10, and 20 days of exposure and gall index (GI) of cucumber roots three weeks after inoculation with J2 treated with the respective AITC concentrations.

Exposure Period	AITC µmol/mL	N (%)	A (%)	I (%)	LC_50_ (µmol/mL)	GI
Day 1	control	93.7	4.7	1.7	0.15 (0.061–0.369)	6.00 ± 0 ^a^
	0.01	89.7	9.3	1.0		5.50 ± 0.5 ^a^
	0.1	38.3	58.0	3.7		4.25 ± 0.5 ^b^
	1	1.0	89.7	9.3		3.00 ± 0.8 ^c^
	5	0.0	34.7	65.3		0 ^d^
	10	0.0	6.3	93.7		0 ^d^
	20–40	0.0	0.0	100		0 ^d^
Day 5	control	92.7	3.3	4.0	0.19 (0.07–0.519)	6.00 ± 0 ^a^
	0.01	74.3	20.7	5.0		5.75 ± .05 ^a^
	0.1	69.3	25.7	5.0		4.75 ± 0.5 ^b^
	1	19.3	45.3	35.3		2.50 ± 0.5 ^c^
	5	0.0	16.0	84.0		0 ^d^
	10	0.0	3.7	96.3		0 ^d^
	20–40	0.0	0.0	100		0 ^d^
Day 10	control	91.3	4.0	4.7	0.10 (0.036–0.335)	5.50 ± 0.5 ^a^
	0.01	64.3	24.0	11.7		5.00 ± 0.8 ^a^
	0.1	60.0	30.0	10.0		4.75 ± 0.5 ^a^
	1	5.7	33.3	61.0		2.50 ± 0.5 ^b^
	5 to 40	0.0	0.0	100		0^c^
Day 20	control	73.7	15.3	11.0	0.07 (0.025–0.184)	4.00 ± 0.8 ^a^
	0.01	55.3	29.3	15.3		3.75 ± 0.5 ^a^
	0.1	46.3	36.7	17.0		3.50 ± 0.5 ^ab^
	1	4.3	32.7	63.0		2.50 ± 0.5 ^b^
	5 to 40	0.0	0.0	100		0 ^c^

Normal (N), affected (A), and immotile (I) second-stage juveniles (J2) are displayed in percentage (%) (*n* = 3). Data for A and I J2 were pooled and log-transformed for LC_50_ (Lethal concentration, 50%) analysis using a linear regression model. Exposed J2 were inoculated on *Cucumis sativus* seedlings and root gall indexing (GI) according to Zeck [24] was determined three weeks later (*n* = 4). Means followed by different superscript letters within the same column indicate significant differences calculated using a one-way ANOVA with post-hoc Tukey HSD test. *p* < 0.05.

**Table 4:** Impact of different sand:soil compositions and organic potting substrate on the efficacy of allyl isothiocyanate (AITC) to reduce the gall index* caused by *Meloidogyne hapla* on *Cucumis sativus*.

AITC µmol/mL	Sand	Sand:Soil Mix			Soil	Potting Substrate
		2:1	1:1	1:2		
Control	5 ± 0.7 ^a1^	5 ± 0.7 ^a1^	5.6 ± 0.5 ^a1^	5.2 ± 1.0 ^a1^	4.8 ± 1.0 ^a1^	5 ± 0.7 ^a1^
1	3.2 ± 0.8 ^b1^	3.6 ± 0.8 ^ab12^	4.2 ± 0.4 ^b13^	4.6 ± 0.5 ^a23^	5 ± 0.7 ^a3^	5.2 ± 0.4 ^a3^
5	2.2 ± 0.8 ^b1^	3.4 ± 0.5 ^b2^	4.2 ± 0.4 ^b23^	4 ± 0.9 ^a23^	4.8 ± 0.4 ^a3^	5.2 ± 0.4 ^a3^
10	0.6 ± 0.8 ^c1^	2.6 ± 0.8 ^bc2^	3.4 ± 0.5 ^bc23^	4 ± 0.7 ^a234^	4.4 ± 0.8 ^ab34^	5.4 ± 0.5 ^a4^
20	0 ^c1^	2.6 ± 0.8 ^bc2^	3 ± 0.7 ^cd23^	3.6 ± 0.8 ^ab234^	4.4 ± 1.1 ^ab34^	5 ± 0.7 ^a4^
40	0 ^c1^	1.2 ± 0.4^c2^	2 ± 0.7 ^d23^	2.25 ± 0.5 ^b234^	3 ± 0.7 ^b34^	5 ± 0.7 ^a5^

* Gall index on a 0–10 scale according to Zeck [24]. Means followed by different superscript letters within the same column indicate significant differences. Means followed by different numbers within the same row indicate significant differences calculated using a one-way ANOVA with post-hoc Tukey HSD test (*n* = 5). *p* < 0.05.

**Table 5:** Effect of allyl isothiocyanate supplementation on the efficacy of the biofumigation potential of three cover crops to control *Meloidogyne hapla*.

	*Meloidogyne hapla* Juveniles (J2/100 mL soil) after AITC Treatment (µmol/mL)					J2 Reduction *
**Cultivars**	0	10	20	40	60	%
***Brassica juncea* cv Terrafit**	85.7 ± 6.6 ^a^	84.5 ± 8.5 ^a^	84.3 ± 8.3 ^a^	74.7 ± 8.1 ^a^	57.1 ± 11.6 ^a^*	33.4
***Raphanus sativus* cv Defender**	81.2 ± 7.7 ^a^	78.7 ± 5.8 ^a^	84.3 ± 7.1 ^a^	83.5 ± 6.7 ^a^	78.5 ± 5.6 ^b^	3.3
**Cover crop mix**	92.2 ± 8.2 ^a^	-	94.5 ± 5.8 ^a^	94.4 ± 5 ^a^	93.6 ± 5.7 ^c^	0.0
**Fallow**	36.3 ± 10.2 ^b^	-	-	-	-	

Means followed by the same superscript letter within a column indicate significant differences between the crops. The asterisks indicate significant differences between different allyl isothiocyanate concentration within a crop calculated using a one-way ANOVA with post-hoc Tukey HSD test (*n* = 6). *p* < 0.05. * J2 reduction by comparison between 0 and 60 µmol/mL AITC treatment–no data available.

## Supplementary Materials

The following are available online at https://www.mdpi.com/2223-7747/9/5/603/s1. Table S1: *Brassicaceae* biomass production including the glucosinolate (GSL) content and quantity (µmol/g dry matter) in the tissue content of the shoots produced by eight cultivars of the species *Brassica juncea* (BJ), *Sinapis alba* (SA) and *Raphanus sativus* (RS), Table S2: Average fresh weight (FW) biomass production in kg/0.2 m^2^ by *Raphanus sativus* (cv. Defender), *Brassica juncea* (cv. Terrafit) and the cover crop UFA Maislegum mix (containing *Trifolium hybridum*, *Medicago lupulina, Trifolium incarnatum,* and *Trifolium repens*) produced during the greenhouse biofumigation tray experiment.
