# Supplementary material for: New Insights on the Role of Allyl Isothiocyanate in Controlling the Root Knot Nematode Meloidogyne hapla
Source: Plants (Basel). 2020 May 9;9(5):603. doi: 10.3390/plants9050603 (PMC7285041; doi:10.3390/plants9050603)
Supplement: Supplementary file 1 [file plants-09-00603-s001.pdf]

Supplementary Table 1. *Brassicaceae* biomass production including the glucosinolate (GSL) content and quantity (µmol/g dry matter) in the tissue content of the shoots produced by eight cultivars of the species *Brassica juncea* (BJ), *Sinapis alba* (SA) and *Raphanus sativus* (RS).

Cultivar		FW (kg/m2)	DW (t/ha)	SIN	RAPH E	ER Y	SIN A	4-OH	GTP	4-MTB	GBC	4-OCH3	NEO	uGSL	GSL (µmol/g DW)	GSL (mol/ha)
BJ	Energy	20.7 ± 4.6	3.42 ± 0.7	11.97	-	-	-	0.23	-	-	0.06	0.73	-	-	12.99	40.9
	Terrafit	21.5 ± 5.2	2.84 ± 0.6	12.85	-	-	-	0.26	-	-	0.16	0.22	-	-	13.50	36.4
	Terraplus	16.3 ± 1.7	2.75 ± 0.3	9.33	-	-	-	0.42	-	-	0.19	0.17	-	-	10.12	25.7
SA	Luna	20.7 ± 2.5	3.89 ± 0.4	-	-	-	11.99	0.04	0.53	-	0.09	0.20	0.03	-	12.88	50.1
	Accent	17.2 ± 0.7	3.18 ± 0.2	-	-	-	8.93	0.03	1.03	-	0.03	0.13	0.01	-	10.16	32.3
RS	Defender	24.9 ±2.9	2.85 ± 0.2	-	1.63	2.69	-	0.30	-	9.98	0.89	0.30	-	3.08	18.88	53.8
	Adagio	27.6 ± 2.9	2.77 ± 0.4	-	1.52	2.33	-	0.11	-	9.82	0.27	0.21	-	3.39	14.25	39.5
	Colonel	29.5 ±4.7	3.48 ± 0.5	-	4.40	6.55	-	0.20	-	3.66	0.76	0.32	-	1.28	17.16	59.7

Fresh weight = FW; dry weight = DW; ha= hectare. Dry matter content of glucosinolate Sinigrin (SIN), Raphenin (RAPHE), Erysolin (ERY), Sinalbin (SINA), Glucotropaeolin (synonym: benzylglucosinolate, GTP), 4-Methylthiobutenyl (4-MTB) Glucobrassicin (GBC), 4-Methoxyglucobrassicin (4-OCH3), Neoglucobrassicin (NEO) and unknown glucosinolate (uGSL) presented in µmol/g (n= one pool of 10 randomly selected plants for each cultivar).

Supplementary Table 2. Average fresh weight (FW) biomass production in Kg/0.2m^2 by *Raphanus sativus* (cv. Deffender), *Brassica juncea* (cv. Terrafit) and the cover crop UFA Maislegum mix (containing *Trifolium hybridum*, *Medicago lupulina, Trifolium incarnatum* and *Trifolium repens*) produced during the greenhouse biofumigation tray experiment.

	Allyl isothiocyanate concentration supplementation (µmol/mL)					
Cultivars	0	10	20	40	60	Average FW in Kg/0.2m^2
Terrafit	2.07 ± 0.05	2.13 ± 0.05	2.25 ± 0.19	2.12 ± 0.20	2.23 ± 0.10	2.16
Defender	2.43 ± 0.21	2.41 ± 0.09	2.52 ± 0.18	2.30 ± 0.15	2.54 ± 0.12	2.44
Maislegum	0.4 ± 0.04	-	0.44 ± 0.04	0.34 ± 0.08	0.42 ± 0.07	0.4

Data are means of six replicates.
